# Supplementary figures and images for: Optimization of Fermentation Conditions and Product Identification of a Saponin-Producing Endophytic Fungus
Source: Microorganisms. 2023 Sep 16;11(9):2331. doi: 10.3390/microorganisms11092331 (PMC10535331; doi:10.3390/microorganisms11092331)

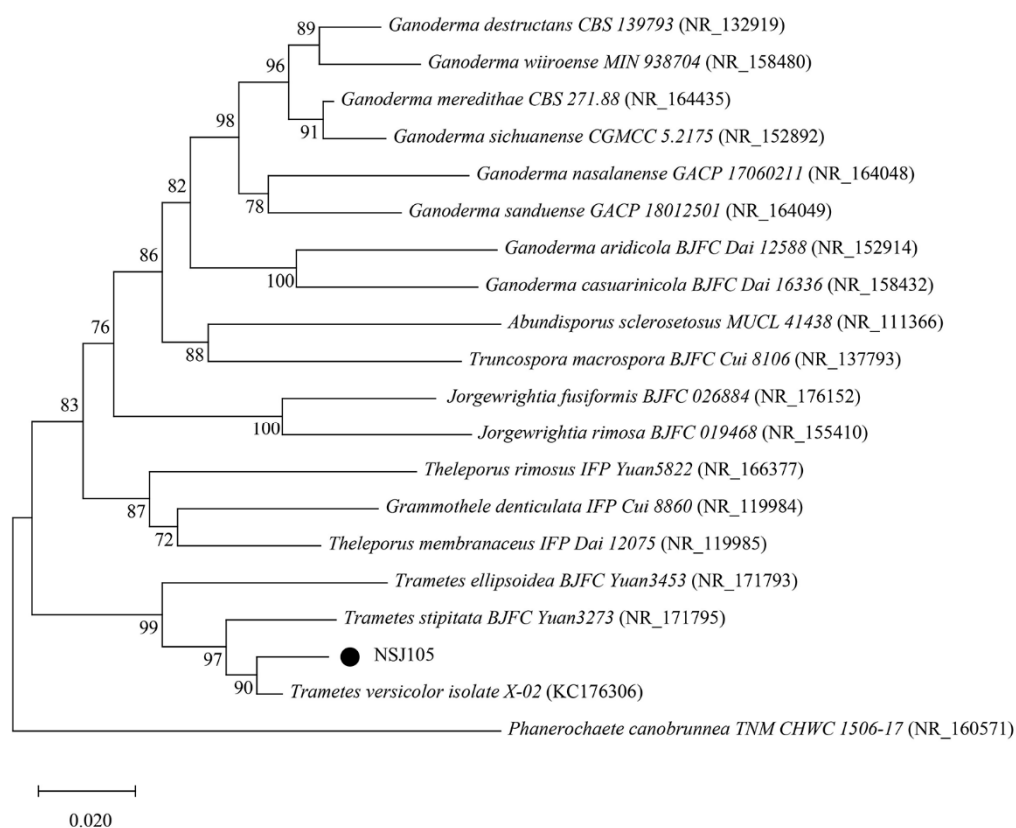

**Figure S1.** Phylogenetic tree characterization of endophytic fungus NSJ105.

Supplement: Supplementary file 1 [file microorganisms-11-02331-s001.zip › Figure S1.pdf]

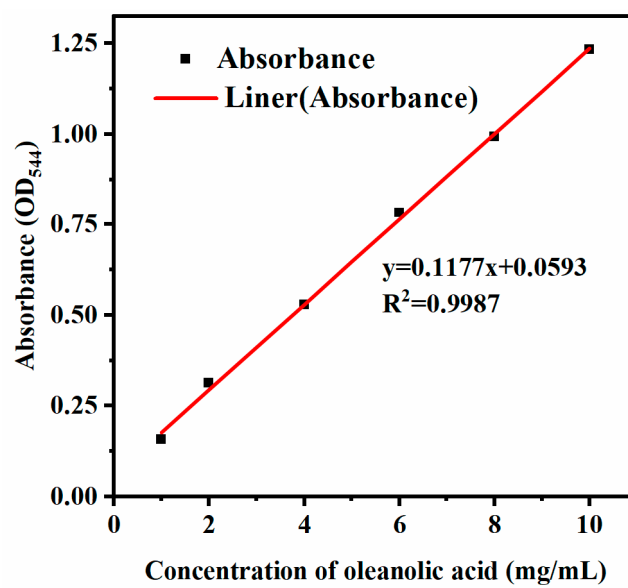

Figure S2. Standard curve for calculation of concentration of total saponins.

Supplement: Supplementary file 1 [file microorganisms-11-02331-s001.zip › Figure S2.pdf]
